# Review on infection control strategies to minimize outbreaks of the emerging pathogen *Elizabethkingia anophelis*

**DOI:** 10.1186/s13756-023-01304-1

**Published:** 2023-09-08

**Authors:** Lisa Mallinckrodt, Robert Huis in ’t Veld, Sigrid Rosema, Andreas Voss, Erik Bathoorn

**Affiliations:** 1 Department of Medical Microbiology and Infection Prevention, University of Groningen, University Medical Center Groningen, Groningen, The Netherlands; 2 Department of Medical Microbiology and Infection Prevention, Gelre Hospital, Apeldoorn, The Netherlands

**Keywords:** *Elizabethkingia anophelis*, Healthcare-associated outbreak, Nosocomial infection

## Abstract

**Background:**

*Elizabethkingia anophelis* is a multi-drug resistant emerging opportunistic pathogen with a high mortality rate, causing healthcare-associated outbreaks worldwide.

**Methods:**

We report a case of *E. anophelis* pleuritis, resulting from transmission through lung transplantation, followed by a literature review of outbreak reports and strategies to minimize *E. anophelis* transmission in healthcare settings.

**Results:**

From 1990 to August 2022, 14 confirmed *E. anophelis* outbreak cohorts and 21 cohorts with suspected *E. anophelis* outbreaks were reported in literature. A total of 80 scientific reports with recommendations on diagnostics and infection control measures were included and summarized in our study.

**Conclusion:**

Strategies to prevent and reduce spread of *E. anophelis* include water-free patient rooms, adequate hygiene and disinfection practices, and optimized diagnostic techniques for screening, identification and molecular typing.

**Supplementary Information:**

The online version contains supplementary material available at 10.1186/s13756-023-01304-1.

## Background

*Elizabethkingia anophelis* is an emerging opportunistic pathogen that has caused several outbreaks in hospitals and health-care facilities around the world in recent years [1–6]. As of today, the largest outbreak has been reported in the Midwestern United States, with a confirmed number of 65 infected patients, of which 20 people deceased. After this outbreak the CDC issued a nationwide alert, followed by a temporary nationwide obligation to report any *Elizabethkingia* species isolate to the CDC [7, 8].

The genus *Elizabethkingia* has first been described in 2005. Two former members of the *Chryseobacterium* genus, namely *C. meningosepticum* and *C. miricola*, were shown through 16 S rRNA gene sequencing to represent a separate lineage within the family *Flavobacteriaceae* and consequently renamed *Elizabethkingia.* [9] *E. anophelis* was first isolated from the midgut of the *Anopheles gambiae* mosquito in 2011 [10]. The new species *E. endophytica* was introduced in 2015, but soon after recognized as *E. anophelis* through whole genome sequencing (WGS) [11]. As of today there are six recognized species in the genus *Elizabethkingia*: *E. meningoseptica*, *E. miricola, E. anophelis*, *E. bruuniana*, *E. ursingii* and *E. occulta* [12, 13].

Members of the *Elizabethkingia* genus are aerobic gram-negative, non-motile rods. *E. anophelis* colonies are smooth, yellowish, translucent, and shiny. They are catalase- and oxidase positive. Unlike other *Elizabethkingia* species, *E. anophelis* does not grow on MacConkey agar [10]. As a result of inconsistent phenotypic characteristics between different species and misidentification using API/ID32 phenotyping, Phoenix 100 ID/AST, VITEK-2, and matrix-assisted laser desorption ionization time-of-flight mass spectrometry (MALDI-TOF MS) systems, *E. anophelis* isolates have often been mistaken for *E. meningoseptica.* [14–19] Since 2017, MALDI-TOF MS systems are able to correctly identify *E. anophelis* isolates [20, 21].

*Elizabethkingia anophelis* has been implied as the causative pathogen in neonatal meningitis, (catheter-related) bacteremia and pneumonia, and are associated with high mortality rates ranging from 18% up to 70% [4, 5, 14, 22]. Treatment of infections with antimicrobial therapy is challenging: *E. anophelis* is a multidrug-resistant bacterium that harbors resistance genes against multiple antibiotic drug classes, such as beta-lactams including carbapenems, aminoglycosides, tetracyclines, fluoroquinolones, macrolide/lincosamide/streptogramins, glycopeptides, folate pathway inhibitors, rifampicin and chloramphenicol [2, 23–25]. Susceptibility rates are highest for minocycline (> 98%), followed by doxycycline (83–92%), piperacillin/tazobactam (27–92%), levofloxacin (16–79%) and trimethoprim-sulfamethoxazole (4–92%) [15, 20, 26]. Furthermore, this micro-organism is difficult to eradicate in the environment, as it can survive in chlorinated water [27]. The possibility of forming a strong biofilm contribute to the pathogenesis and resilience of this micro-organism [28].

Given the high mortality rates of infected patients, the limited therapeutic options and the probability of nosocomial outbreaks, *E. anophelis* is a bacterium of great concern. Optimization of detection methods and infection control measures are necessary to minimize future nosocomial outbreaks by *E. anophelis*. In this article we describe a case of *E. anophelis* pleuritis transmitted through bilateral lung transplantation, followed by a review of the literature on healthcare-associated *E. anophelis* outbreaks, and provide recommendations on infection prevention strategies and control measures based on the published scientific evidence and our own experience.

## Case presentation

A 61-year-old man with severe pulmonary emphysema received a bilateral lung transplant from a non-heart-beating donor in July 2021. Inspection of the lungs including bronchoscopy during the procurement procedure did not show any irregularities. The lungs were transplanted to the patient without the need of extracorporeal circulation. The patient was extubated according to protocol after inspection by bronchoscopy on the first day after surgery.

**Fig. 1:**
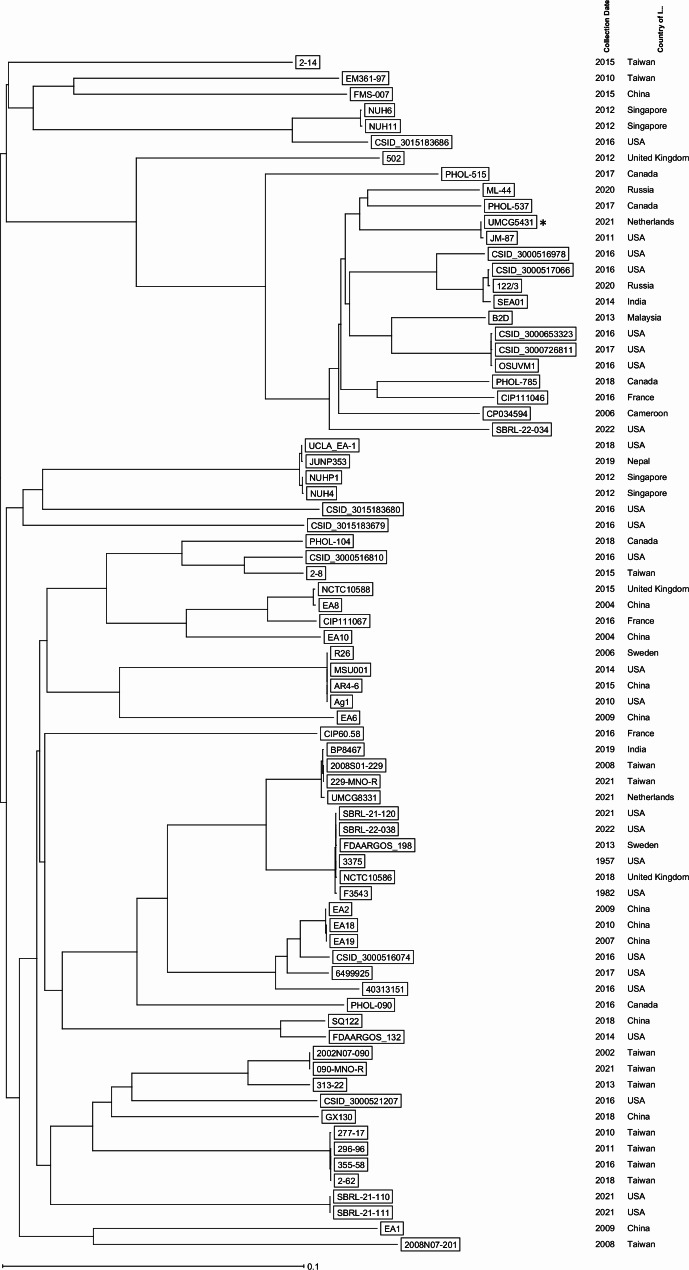
Neighbor joining phylogenetic tree based on single nucleotide variants (SNV) The isolate from our case is marked with an asterisk

Respiratory secretions obtained from the donor lung prior to transplantation and by bronchoscopy on the first day after transplantation initially only grew *Haemophilus influenzae* and methicillin-susceptible *Staphylococcus aureus*. On the fifth day after transplantation the right thoracic drain was removed and cultured on 5% sheep blood agar (BA) plates at 35˚C O_2_ for 48 h and on and MacConkey agar (MAC) plates at 35˚C CO_2_ for 48 h. Grey colonies were visible on BA, which were identified as *E. anophelis* by MALDI-TOF MS (MALDI Biotyper v9.0, Brucker Daltonics, Bremen, Germany). No growth was seen on MAC. The cultures were found to be positive for *E. anophelis*. 16 S- and SNP-based molecular analysis of the whole genome sequence of this isolate was performed as described previously, confirming the species determination of *E. anophelis* (Fig. 1) [29].

Antibiotic susceptibility was tested by gradient strips (Etest®; bioMérieux S.A., Marcy l’Etoile, France) on Mueller Hinton Agar, by broth microdilution test (Sensititer, Thermo Fisher Scientific, Waltham, MA,) and by automated susceptibility testing (BD Phoenix, Sparks, MD). The isolate was susceptible only to trimethoprim/sulfamethoxazole (1 mg/L), minocycline (0.25 mg/L) and doxycycline (1 mg/L) but resistant to all other drugs tested including ampicillin, amoxicillin/clavulanic acid, ceftriaxone, ceftazidime, cefepime, aztreonam, imipenem, amikacin, tobramycin, and colistin. Very major discrepancies were observed for ciprofloxacin and moxifloxacin between susceptibility methods. In such cases the result of broth microdilution was leading. The susceptibility pattern was consistent with existing literature [15, 16, 20]. Further information regarding susceptibility results can be found in Supplementary Table 1.

Because of the multidrug resistant nature of *E. anophelis* and its propensity for nosocomial spread, the patient was placed in contact isolation measures immediately after identifying the isolate. As part of source detection, frozen respiratory samples from the donor lung were thawed and cultured again, this time on *Burkholderia cepacia* selective agar (BCSA) containing gentamicin, vancomycin, and polymyxin B sulphate (Mediaproducts BV, Groningen, The Netherlands), revealing the presence of *E. anophelis* in two samples. These findings suggest that *E. anophelis* had been introduced via the donor lung, and most probably spread to the pleural cavity as a result of leakage or spill during surgery. Screening cultures of rectal swabs and throat swabs of three close contact patients using BCSA were negative. The hospital where the donor lungs were harvested was notified of our finding. Contact isolation precautions were maintained until three consecutive sputum samples were negative. These samples were collected during a second period of hospitalization two months after the last positive cultures, on three separate days with one day in between each day. The patient did not receive any antibiotic therapy when the follow-up samples were collected.

The patient was treated with a combination of trimethoprim/sulfamethoxazole 960 mg twice daily and minocycline 100 mg twice daily. Despite prompt treatment, there was an increase in CRP levels (up to 100 mg/L), leukocyte count (20.6*10^9/L) and pleural effusion in the second week after surgery. There was no fever. The inflammatory parameters slowly decreased after five days of antibiotic therapy. Nevertheless, cultures of the fluid from the second right thoracic drain remained positive until removal of the drain on day 16 post-transplantation. Culture of this drain tip also revealed *E. anophelis*. Antibiotic treatment with trimethoprim/sulfamethoxazole and minocycline was discontinued two weeks after all drains were removed. The patient was discharged in good condition on day 33 after surgery. Cultures were negative during two months follow up after transplantation.

## Literature review

### Search strategy and selection criteria

**Fig. 2:**
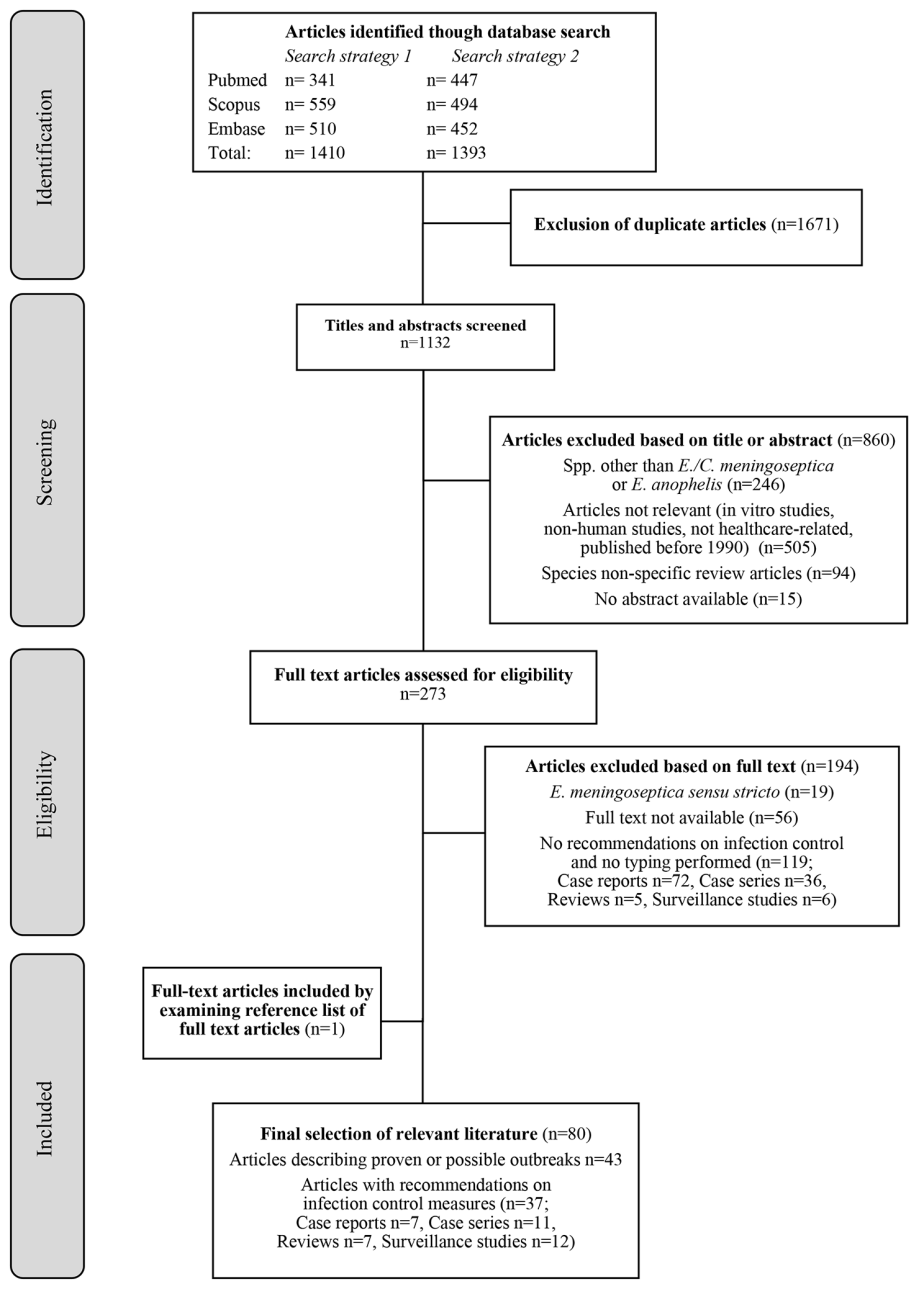
Study selection

A literature search was performed on March 18, 2022 with the following search terms in Pubmed “*(Elizabethkingia[title/abstract] OR Chryseobacterium[title/abstract])*” (filters applied: English language, human studies), in Scopus “*TITLE-ABS-KEY (elizabethkingia OR chryseobacterium) AND (LIMIT-TO (SUBJAREA, “MEDI”)) AND (LIMIT-TO (LANGUAGE, “English”) AND (LIMIT-TO (EXACTKEYWORD, “Human”))*”, and in Embase “*(((elizabethkingia:ab,ti OR chryseobacterium:ab,ti) AND english:la) AND ‘human’/de)*”. On August 12, 2022 an additional search was performed to include studies that were missed in the first search. The following search terms were used: “*(elizabethkingia[title/abstract] OR “chryseobacterium meningosepticum“[title/abstract])*” in Pubmed, “*TITLE-ABS-KEY (elizabethkingia OR “chryseobacterium meningosepticum”) AND (LIMIT-TO (SUBJAREA, “MEDI”)) AND (LIMIT-TO (LANGUAGE, “English”))*” in Scopus, and “E*lizabethkingia:ab,ti OR ‘Chryseobacterium meningosepticum’:ab,ti*” in Embase. Only full text articles describing outbreaks or recommendations for diagnostics or infection control were included in the final selection. Studies on *E. anophelis* identified by molecular methods or by MALDI-TOF MS after 2017 were included as confirmed outbreak cases. Molecular identification before 2017 is less reliable, since the 16 S rRNA of *E. anophelis* and *E. meningoseptica* are 99% similar, which have caused misidentified species in reference databases [30]. *Elisabethkingia/Chryseobacterium* species with no growth on MAC before 2017 were included as possible *E. anophelis* outbreak cases. Studies published prior to 1990 were excluded (Fig. 2).

## Results

Up to March 2022, 20 studies with results from environmental culturing and/or genotyping (outbreak reports) were published related to 14 cohorts with confirmed *E. anophelis* cases (Table 1).

**Table 1:** Overview of included articles with confirmed *E. anophelis* infections

Outbreak number	Period	Country	Number of infected patients	Setting	Typing performed	Environmental surveillance performed	Reference
1	Sept 2020 – Sept 2021	France	20	healthcare facilities	yes	yes	[31]
2	July - Sept 2020	Argentina	9	neonatal unit	yes	yes	[32]
3	2017–2018	Taiwan	20*	hospital wards	yes	no	[33]
4	Aug - Sept 2017	India	9*	hospital wards	yes	no	[18]
5	May 2017	Singapore	3	pediatric ICU	no	yes	[34]
6	Jan 2016 - June 2017	South Korea	79	ICU, hospital wards	yes	yes	[35]
7	June 2014 - March 2016	Illinois, USA	10	healthcare facilities	yes	no†	[5]
8	Nov 2015 - June 2016	Wisconsin, USA	63	health care facilities	yes	yes	[3, 4, 8, 36]
9	2015–2018	Taiwan	26	respiratory care center	yes	yes	[6, 37]
10	2012–2018	Australia	14	hospital ward	yes	yes	[21]
11	Aug - Sept 2012	Singapore	5	ICU	yes	yes	[2, 38, 39]
12	2012–2018	Shanghai, China	35	hospital wards	yes	no	[40]
13	2005–2016	Taiwan	67	hospital wards	yes	no	[26]
14	2004–2013	Hong Kong	17*	hospital wards	yes	no	[14]

* Only patients with bacteremia were included

† Outbreak cluster was related to non-specified environmental isolates from previous outbreak in 2012–2013

Additionally, there were 22 outbreak reports related to 21 patient cohorts in which the causative pathogen was probably *E. anophelis*. (Supplementary Table 2). Taken together, 35 outbreaks with *E. anophelis* have been reported from hospitals on all continents, and the majority of outbreaks were reported from Taiwan (n = 11), India (n = 5), and the United States (n = 5). The outbreaks by *E. anophelis* have taken place in both adult and pediatric wards or ICUs. Environmental surveillance was performed in 8 of 13 confirmed *E. anophelis* outbreaks (Table 2). In cohorts with positive environmental cultures, water points were most commonly identified as the source of the outbreak. Genotyping was performed in 13 of the 14 confirmed *E. anophelis* outbreak cohorts included in our review. All these studies reported clusters of isolates identified by molecular typing methods such as RAPD, rep-PCR, PFGE, and WGS (Table 3). The outbreak numbers in Tables 2 and 3 correspond with the outbreak numbers in Table 1.

Genetically related isolates were not always geographically related, and some completely identical isolates were found in different countries which implies a different route of transmission. For example, in one incident the international export of medical equipment contributed to the worldwide spread of *E. anophelis* through contaminated commercial SARS-CoV-2 swab kits [41].

**Table 2:** Environmental surveillance results of outbreaks with confirmed *E. anophelis*

Outbreak number*	Environmental surveillance	Surveillance culture method	Positive environmental cultures	Environmental isolate related to outbreak cluster	Source	Possible transmission route
1	Hospital water from central tank and wards, dialysis water, dialysis fluid, seven bottles of disinfectant, oxygen masks, distributed oxygen gases, community tap water and a rainwater cistern	Columbia agar	none	-	Unknown	Unknown
2	50 cultures from 25 potential surfaces and equipment including incubators, monitors, sinks, faucets, aerators and water from faucets.	n/a	Faucet aerator in material washing waste basin (n = 1)	Yes	Faucet aerator	Water
5	27 cultures from 9 water points: sinks, aerator swabs and water samples.	n/a	2 water samples, 4 aerators, 3 sinks (n = 9)	Typing not performed	Water taps	Health care worker’s hands
6	281 swab cultures of equipment and surfaces within patient rooms, restrooms, nursing stations, electronics, furniture, patient care devices, patient transport carts, sinks, and water taps.	BA and MAC, 24 h	4 water taps in ICU, 2 washbasins in ICU, 1 suction regulator in hospital ward (n = 7)	Yes, all 7 environmental isolates in Cluster 1	Cluster 1: water taps Other clusters: unknown	Water
8	41 cultures of healthcare and personal care products, 29 samples of tap water 61 water-associated biofilm samples (n = 131)	n/a	1 sample of standing water with contaminated patient material	Typing not performed	Unknown	Unknown
9	34 tap water samples and 117 surface swabs (beds, monitors, remote controllers, light switches feedings tubes/bags swabs and sputum suction regulator swabs)	Tryptic soy broth, 48 h followed by BA and MAC 18-24 h CO_2_	18 tap water samples and 4 surface swabs (2 feedings tubes/bags and 2 sputum suction regulators) (n = 22)	Yes, 4/4 surface swabs and 5/14 water samples were related (PFGE). Typing not performed on remaining 4 water samples.	Water taps	Feeding tube/bag and sputum suction devices
10	n/a	5% horse agar	3 sinks, 2 sink drains and one handrail (n = 6)	Yes, 2 sink swab related to outbreak isolates. Other 4 isolates not related.	Sink	Unknown
11	15 swabs of equipment or re-usable items, 79 aerator swabs, swabs of internal surfaces of 5 water taps, 10 samples of dialysis water in patient rooms, 8 samples from dialysis taps in dialysis centres, 6 aerators in OR scrub rooms, unknown number of water samples of central water supply.	BA, 48 h	35 aerator swabs, 5 swabs of internal surface of water taps.†	14/14 aerator swabs were > 99% similar to clinical isolates (Rep-PCR) 2/4 aerator swabs < 180 SNP difference (WGS)	Water taps	Water

* Outbreak numbers correspond with outbreak numbers in Table 1. †All clinical and environmental isolates were (mis)identified as *E. meningoseptica*, but correctly identified as *E. anophelis* through WGS on available clinical isolates (n = 3) and environmental isolates (n = 4)

n/a = not available, BA = blood agar (tryptic soy agar + 5% sheep blood), MAC = MacConkey agar

**Table 3:** Diagnostic methods and typing results of proven outbreaks

Outbreak number*	Method of detection in clinical samples	Method of species determination	Typing method	Number of clinical isolates typed	Cluster size	Cluster definition
1	n/a	MALDI-TOF, WGS	WGS	20	20	n/a *Isolate difference 4–28 SNPs*
2	KPC CHROMagar (rectal swabs n = 6) or n/a (n = 3)	MALDI-TOF, 16 S	WGS	9	8	n/a
3	n/a	MALDI-TOF	RAPD-PCR	20	Cluster 1/2/3: 3 Cluster 4/5/6: 2	> 85% similarity
4	n/a	VITEK-2, 16 S†	Rep-PCR	9	Cluster 1: 3 Cluster 2: 2 Cluster 3: 2	n/a
6	n/a	MALDI-TOF, 16 S	PFGE	40	Cluster V: 25 Cluster VII: 6 Cluster I/III/IV: 2	n/a *Isolates within a cluster were > 88% similar*
7	n/a	WGS	PFGE + WGS	11	10	< 60 SNPs distance by WGS
8	n/a	MALDI-TOF, WGS†	WGS	69	66 Subcluster 1: 13 Subcluster 2: 6/69 Subcluster 3: 9/69 Subcluster 4: 3/69 Subcluster 5: 2/69 Subcluster 6: 26/69	n/a
9	n/a	MALDI-TOF, WGS	PFGE + WGS	26 (PFGE), 18 (WGS)	26 (PFGE), 18 (WGS)	> 80% (PFGE) n/a (WGS)
10	n/a	MALDI-TOF, WGS	WGS	14	2	n/a
11	BA, 36 °C, 48 h	MALDI-TOF, 16 S, WGS†	Rep-PCR, WGS	3	3	n/a *Isolates were > 99% similar (Rep-PCR), or < 30 SNPs distance (WGS)*
12	n/a	16 S, species specific PCR	PFGE	34	Cluster A: 8 Cluster H/I: 4 Cluster D: 3 Cluster F/G/J/K/M: 2	n/a *Isolates were > 85% similar*
13	n/a	16 S	PFGE	66	Cluster 10: 20 Cluster 1: 16 Cluster 12: 7 Cluster 7: 6 Cluster 11: 3 Cluster 4/6: 2	> 85% similarity
14	n/a	MALDI-TOF, 16 S†	PFGE	17	2‡	n/a *Isolates were 93% similar*

*Outbreak numbers correspond with outbreak numbers in Table 1. † Isolates were misidentified by MALDI-TOF MS or VITEK-2, but later correctly identified as *E. anophelis* by 16 S and/or WGS ‡Isolates were obtained from patients in two different hospitals with community acquired pneumonia. n/a = not *available*

### Recommendations for infection prevention and outbreak control

In Table 4, we present a summary of recommendations for infection control and diagnostics to prevent and control *E. anophelis* outbreak along with the references that support the recommendations.

### Prevention of outbreaks

The first set of recommendations focuses on the prevention of outbreaks. Water taps in patient rooms in general, and those with aerators in particular, have most commonly been identified as source of outbreaks. Infection control specialists should be counciled in the design of patient rooms. In high risk units like intensive cares, the use of wet points should be avoided as much as possible. If the use of water can not be avoided, the design should be in such a way that the risks of splashing and contaminating patients, bedding and towels, and medical equipment are minimized. The use of aerators should be avoided. In addition, taps should be flushed daily to avoid colonization of taps in biofilms. The periodical surveillance of watertaps for contamination is important to identify risks in an early stage. Tap water system contamination with gram-negative bacilli (GNB) is associated with patient colonization, and removal of sinks on ICU wards has been proven to reduce the colonization rate of patients with GNB [42, 43]. In a small experiment performed by Yung et al., acquisition of *E. anophelis* through hand washing with chlorhexidine soap and water from a contaminated water source has been proven [34]. It is therefore recommended to aim for water-free patient care, especially in vulnerable populations, and to focus on alcohol rub instead of hand washing with water and soap during hand hygiene procedures. In general, the colonization and infection of patients could be prevented by lowering antibiotic selective pressure through antibiotic stewardship. In populations with high risk of acquisition of highly resistant microorganisms due to increased antibiotic use such as in the ICU, it is recommended to screen patients for colonization with GNB in sputum, throat swabs and rectal swabs. It is recommended to collect antimicrobial resistance data including the *E. anophelis* prevalence in a national surveillance program. Such a database could be consulted when confronted with an unexpected finding. Since *E. anophelis* has scarcely been detected in other Dutch hospitals, there was no indication of an inter-hospital outbreak.

### Outbreak control

In the second set of recommendations in Table 4, we focus on outbreak control. In outbreak management, it is important to conduct source investigation and contact tracing, including environmental cultures, water samples and testing of close contacts. Changes of care providers should be restricted until the source of the outbreak is found. In most of the clusters described in the literature, contaminated water points have been identified as the source of the outbreak. Such sources should be eliminated as soon as possible to control the outbreak. The contaminated water source can also be outside of the hospital: several outbreaks have been reported in Taiwan, possibly introduced into healthcare settings after the Formosa Fun Coast dust explosion where burn victims were cooled with pool water [44].

In addition to waterpoints, transmission through ERCP and mother-to-infant transmission have been described [45, 46]. Infections derived from donors have been identified in two patients who underwent transplantation of tendon-bone and ligament allografts. The likely cause of contamination was during the processing stage as the organism was found in the sink drains and traps in the clean processing rooms [47]. Unlike in our case, in other reported cases of *Elizabethkingia* spp. infections after solid organ transplantation the source or transmission route have never been identified [48].

In order to prevent donor-transmitted bacterial pneumonia, lung transplant recipients are treated with a broad-spectrum antibiotic, which is modified on the basis of cultures obtained from the donor lungs [49]. In our medical center we culture sputum from donor lungs on BA (ambient air, 35 °C, 48 h), CHOC and MAC (both 5% CO_2_, 35 °C, 48 h), and on Sabouraud agar with aztreonam and vancomycin (ambient air, 35 °C, 5 days and 28 °C, 4 weeks). With this screening protocol *E. anophelis* can easily be missed in the cultured flora on non-selective BA and CHOC. In order to be able to selectively detect *E. anophelis*, BCSA was shown useful.

Patients that are positive for *E. anophelis* should be placed within barrier precautions to prevent patient-to-patient transmission. In addition, the disinfection of the patient environment should be enhanced. Chlorine-disinfectants are reported to be insufficient against *E. anophelis.* [27, 39, 50] Disinfection with hydrogen peroxide-based agents has been recommended as an adjunctive measure [51]. In our hospital, we use hydrogen-peroxide based wipes (Incidin™ OxyWipe, Ecolab, The Netherlands) to disinfect small surfaces and equipment, and a hydrogen-peroxide based solution (Terralin© PAA, Schülke & Mayr, Germany) to disinfect larger contaminated patient areas.

### Typing methods

To detect and characterize an outbreak, molecular typing should be performed. The typing results provide information if there is clonal transmission of a strain, or if multiple clones from potentially different sources are involved. For instance, the typing results of the largest described outbreak in an ICU in a hospital in South Korea which inclused 79 confirmed cases showed that there had been transmission of multiple different clones [35].

Typing results can be challenging to interpret. Cut-off values for typing are not well-established and range from 80 to 93% in PFGE in our literature search. For WGS there are no standardized cut-off points to identify clusters: in the study by Navon et al., < 60 SNPs was chosen as the cut-off value to discriminate isolates from each other [5]. Genetic distance is impacted by pre-existing diversity in the source host, plus the amount of SNPs that accumulates in the source and recipient hosts over time [52]. Since genomic instability is species-specific cutoff values cannot be extrapolated by default. To determine a cut-off value it is therefore essential to sequence a large collection of isolates, which is a challenge with infrequently cultured micro-organisms. Compared to PCR-based typing methods, genome sequencing has a greater discriminatory power and provides more information regarding the phylogeny [53]. Isolates belonging to the same PFGE patterns can have variable resistance profiles [54]. This could be either attributed to unreliable resistance profiling, or to insufficient discriminatory value of PFGE typing. The higher discriminatory power and transferability of data makes WGS the typing method of choice whenever possible.

**Table 4:** Recommendations for infection control and diagnostics to prevent and control *E. anophelis* outbreaks

Recommendations		Study
Infection prevention	Involve infection control staff in the early planning stages of designing patient rooms and wards, especially within critical care settings	[32, 38, 39, 55, 56]
	Establish a water management program	[55–59]
	Aim for water-free patient rooms in high-risk populations	[60] [This study]
	Use tap outlets without aerators	[34]
	Flush taps at least daily (automatically)	[6, 50, 55]
	Implement surveillance on water contamination on a periodic basis	[4, 8, 27, 28, 40, 54, 55, 61–65]
	Implement an antibiotic stewardship program	[66–69]
	Implement a national AMR surveillance program	[37, 57, 70]
	Screen high-risk patients and donor tissues for multidrug resistant gram-negative bacilli	[71] [This study]
	Continuously educate and monitor care providers on hand hygiene, disinfection practices and aseptic technique	[6, 27, 32, 34, 42, 54, 55, 58, 59, 61, 63, 66, 68, 69, 72–79]
	Use alcohol-based hand rub instead of water and soap based hand hygiene	[6, 34, 50, 60]
	Discard baby pacifiers (dummies) every 24 h	[80–82]
	Avoid contact between clean water points and body fluids and body fluid-contaminated items	[34, 38, 39, 51, 75, 83]
	Use sterile water for patient bathing and cleaning of medical equipment and patient-care items	[2, 6, 34, 38, 39, 42, 62, 72, 75, 76, 83, 84]
Outbreak control	Isolate patients with positive *Elizabethkingia* cultures	[4, 8, 58, 61, 79, 85–87] [This study]
	Conduct source investigation and contact tracing, including environmental cultures, water samples and testing of close contacts	[4, 8, 42, 51, 58, 59, 66, 79, 88–91] [This study]
	Use selective media such as Burkholderia Cepacia Selective Agar or combined disc tests for screening purposes	[33] [This study]
	Enhance disinfection of surfaces and equipment during an outbreak	[4, 6, 8, 32, 34, 35, 40, 50, 51, 61, 63, 64, 66, 68, 72, 74, 79, 87–89, 92]
	Restrict staff exchange during an outbreak until source is found	[61, 92]
	Remove or replace contaminated water sources	[6, 32, 38, 39, 50, 63, 66, 83]
	Identify clusters through whole genome sequencing	[2, 3, 5, 17, 93–95]

### Concluding remarks

The transmission described in this study did not lead to further transmission to contacts of the lung transplant recipient. We have notified the transplant coordinator on the positive *E. anophelis* cultures after the lung transplant, since other donated organs may also be contaminated. Because the privacy of donors is strictly protected, we have not been informed on positive cultures in other donated organs, or transmission in the institution of the donor. Unfortunately, the isolate obtained from the donor was no longer available for sequencing to confirm their clonality. Prior to this case *E. anophelis* was cultured only once in our medical center from a deep wound infection in April 2021. This isolate was still available and found not to be related using WGS analysis (42.067 single nucleotide difference, marked as *UMCG 8831* in Fig. 1). Direct transmission from the organ donor to the recipient in our case is therefore the most likely transmission route. Several recommendations were already implemented in our medical center, reducing the likelihood of spread. For instance, our intensive care units are designed without water taps in patient areas. Extensive environmental screening was not performed because it was assumed that the *E. anophelis* was either community acquired or acquired in the donor hospital.

## Conclusion

In conclusion, *E. anophelis* is a multi-drug resistant nosocomial pathogen, as demonstrated by the plentitude of healthcare-related outbreak reports. Surveillance and water management are important measures to prevent large outbreaks. Outbreak investigation should include contact investigations and environmental sampling using selective culturing agars, to find and eradicate a source. The most commonly detected sources of outbreaks were water taps with aerators, however, transmission from patient-to-patient, through contaminated medical equipment or donor tissue as in the presented case are also established routes. Isolates should be typed preferably by WGS to characterize outbreaks, identify clonal transmission and facilitate exchange of genetic data.

### Electronic supplementary material

Below is the link to the electronic supplementary material.

Supplementary Material 1

Supplementary Material 2

## Data Availability

Sequencing data is available from the European Nucleotide Archive, Bioproject PRJEB61750.

## List of abbreviations

BA: Blood agar
BCSA: *Burkholderia cepacia* selective agar
CHOC: Chocolate agar
GNB: Gram negative bacilli
ICU: Intensive care unit
MAC: MacConkey agar
MALDI-TOF: MS Matrix-assisted laser desorption ionization time-of-flight mass spectrometry
SNP: Single nucleotide polymorphism
SNV: Single nucleotide variants
WGS: Whole genome sequencing
