# Supplementary material for: Review on infection control strategies to minimize outbreaks of the emerging pathogen Elizabethkingia anophelis
Source: Antimicrob Resist Infect Control. 2023 Sep 8;12:97. doi: 10.1186/s13756-023-01304-1 (PMC10486102; doi:10.1186/s13756-023-01304-1)
Supplement: Supplementary file 1 — Supplementary Material 1 [file 13756_2023_1304_MOESM1_ESM.docx]

**Supplementary table 1**

| Antimicrobial agent | Breakpoint (mg/L)^a^ | | MIC clinical isolate (mg/L)^b,c^ | | | Interpretation^d^ | MIC range reported in literature (mg/L)^e^ | MIC_50_ reported in literature (mg/L) | MIC_90_ reported in literature (mg/L) | Total number of reported strains | Ref |
| --- | --- | --- | --- | --- | --- | --- | --- | --- | --- | --- | --- |
|  | S ≤ | R ≥ | BM | GS | AST |  |  |  |  |  |  |
| Penicillins | | |  |  |  |  |  |  |  |  |  |
| Ampicillin | 2^E^ | 16^E^ |  |  | >8 | R |  |  |  |  |  |
| Piperacillin | 8^E^ | 32^E^ |  |  | >64 | R | <16->64 | 64 | >64 | 67 | [26] |
| Ticarcillin | 8^E^ | 32^E^ |  |  | >64 | R |  |  |  |  |  |
| β-lactam combinations | | |  |  |  |  |  |  |  |  |  |
| Amoxicillin/  clavulanic acid | 2^E^ | 16^E^ |  |  | >32 | R |  |  |  |  |  |
| Piperacillin/  tazobactam | 16^C^ 8^E^ | 128^C^ 32^E^ |  | 12 | 4 | S/I (MD) | 8-64  ≤8-≥128 <8->128 | 16  16  64 | 16  64  >128 | 79  105  67 | [15]  [20]  [26] |
| Cephalosporins | | |  |  |  |  |  |  |  |  |  |
| Cefepime | 8^C^ 4^E^ | 32^C^ 16^E^ |  |  | >16 | R | 16->16  8->32 | >16  >32 | >16  >32 | 79  67 | [15]  [26] |
| Ceftazidime | 8^C^ 4^E^ | 32^C^ 16^E^ |  |  | >16 | R | >16  >16 | >16  >16 | >16  >16 | 79  67 | [15]  [26] |
| Ceftriaxone | 8^C^  1^E^ | 64^C^ 4^E^ |  |  | >4 | R? | 32->32 | >32 | >32 | 67 | [26] |
| Cefuroxime | 4^E^ | 16^E^ |  |  | >8 | R |  |  |  |  |  |
| Monobactams | | |  |  |  |  |  |  |  |  |  |
| Aztreonam | 8^C^ 4^E^ | 32^C^ 16^E^ |  |  | >16 | R | 8->16  >16 | >16  >16 | >16  >16 | 79  67 | [15]  [26] |
| Carbapenems | | |  |  |  |  |  |  |  |  |  |
| Imipenem | 4^C^ 2^E^ | 16^C^ 8^E^ | >32 |  | >8 | R | 8->8  8->8 | >8  >8 | >8  >8 | 79  67 | [15]  [26] |
| Meropenem | 4^C^ 2^E^ | 16^C^ 8^E^ |  |  |  |  | 8->8  >8 | >8  >8 | >8  >8 | 79  67 | [15]  [26] |
| Aminoglycosides | | |  |  |  |  |  |  |  |  |  |
| Amikacin | 16^C^  1^E^ | 64^C^  2^E^ | 64 |  | >16 | R | 4-32  16->32 | >32  >32 | >32  >32 | 79  67 | [15]  [26] |
| Gentamicin | 4^C^  0.5^E^ | 16^C^ 1^E^ |  |  |  |  | 4->8  8->8 | >8  >8 | >8  >8 | 79  67 | [15]  [26] |
| Tobramycin | 4^C^  0.5^E^ | 16^C^  1^E^ | >16 | >256 | >4 | R | 8->8  >8 | >8  >8 | >8  >8 | 79  67 | [15]  [26] |
| Tetracyclines | | |  |  |  |  |  |  |  |  |  |
| Doxycycline | 4^C^ | 16^C^ | 1 |  |  | S | <2-8  ≤2-8 | <2  4 | 4  8 | 79  105 | [15]  [20] |
| Minocycline | 4^C^ | 16^C^ | 0.25 | 0.38 |  | S | <2->16  ≤2-16  <1-4 | <2  ≤2  <1 | <2  ≤2  <1 | 79  105  67 | [15]  [20]  [26] |
| Tigecycline | 0.5^E^ | 1^E^ | 1 |  |  | R | <0.25->8  1-8  <1->8 | 2  16  4 | 8  64  8 | 79  105  67 | [15]  [20]  [26] |
| Tetracycline | 4^C^ | 16^C^ | 6 |  |  | I | >8 | >8 | >8 | 67 | [26] |
| Fluoroquinolones | | |  |  |  |  |  |  |  |  |  |
| Ciprofloxacin | 1^C^ 0.25^E^ | 4^C^ 1^E^ | 8 | 0.38 | <0.25 | R (VMD) | 0.25->2 1-≥4  <1->32 | >2 ≥4  2 | >2 ≥4  >32 | 79 105  67 | [15]  [20]  [26] |
| Levofloxacin | 2^C^ 0.5^E^ | 8^C^ 2^E^ |  |  | ≤0.5 | S | ≤1->8  ≤1->8  <1->32 | 2  >8  2 | >8  >8  >32 | 79  105  67 | [15]  [20]  [26] |
| Moxifloxacin | 0.25^E^ | 0.5^E^ | 4 | 0.094 |  | R (VMD) |  |  |  |  |  |
| Other agents | | |  |  |  |  |  |  |  |  |  |
| Clarithromycin |  |  | 16 |  |  |  |  |  |  |  |  |
| Clofazimine |  |  | 1 |  |  |  |  |  |  |  |  |
| Colistin |  |  |  |  | >4 |  | >4 | >4 | >4 | 79 | [15] |
| Linezolid | 2^E^ | 4^E^ | 8 |  |  | R |  |  |  |  |  |
| Rifabutin |  |  | 0.25 |  |  |  |  |  |  |  |  |
| Rifampicin |  |  | 0.5 | 0.38 |  |  |  |  |  |  |  |
| Trimethoprim/  sulfamethoxazole^f^ | 2^C^ | 4^C^ | 1 | 0.25 |  | R | 0.5-2  1-4  <2->4 | 1  4  >4 | 2  4  >4 | 79  105  67 | [15]  [20]  [26] |
| Vancomycin |  |  |  | 16 |  |  | 8-64 | 16 | 32 | 67 | [26] |

^a^ C = Clinical and Laboratory Standards Institute (CLSI) for non-Enterobacteriaceae (M100-ED32:2022); E= European Committee on Antimicrobial Susceptibility Testing (EUCAST) non-species-specific breakpoints (V12.0) ^b^ BM = broth microdilution; AST = automated susceptibility testing; GS = gradient strip ^c^ Trailing was observed with moxifloxacin (0.12mg/L to 4 mg/L) and ciprofloxacin (0,5 mg/L to 8.mg/L) ^d^ MD = major discrepancy (susceptibility in broth microdilution but resistance in gradient strip or AST); VMD= very major discrepancy (resistance in broth microdilution but susceptibility in gradient strip or automated susceptibility testing); ^e^  Only susceptibility profiles obtained using microdilution are reported;
^f^ Trimethoprim/sulfamethoxazole in the ratio 1:20. MIC is expressed as the trimethoprim concentration
